# Presentation characteristics and clinical outcome of patients with giant cell arteritis followed by a single center

**DOI:** 10.55730/1300-0144.5391

**Published:** 2021-09-24

**Authors:** Mert ÖZTAŞ, Hamit ÖZGÜL, Emire SEYAHİ, Serdal UĞURLU

**Affiliations:** Division of Rheumatology, Department of Medicine, İstanbul University-Cerrahpaşa, İstanbul, Turkey

**Keywords:** Giant cell arteritis, polymyalgia rheumatica, PET-CT, temporal artery biopsy, headache

## Abstract

**Background/aim:**

Giant cell arteritis (GCA) is a large vessel vasculitis that may cause significant morbidity in the elderly population. We aimed to evaluate presentation characteristics, treatment, and outcome in a cohort of patients with GCA diagnosed and followed in a single center.

**Materials and methods:**

A retrospective chart review revealed 84 (41 M/43 F) registered patients diagnosed with GCA between 1990 and 2020. Clinical features at presentation and follow-up, radiographical imaging, temporal artery biopsy (TAB), and laboratory findings were retrieved from digital medical records or hard-copy patient files. Of these, 33 patients’ follow-up period was less than 12 months; hence, relapses and treatment outcomes were examined in the remaining 51 (60.5%) patients.

**Results:**

A total of 84 patients were included in the cohort. The mean age at diagnosis was 68.4 ± 7.9 years (range: 49–85). At presentation, 60 (71.4%) patients had headache, 22 (26.2%) had symptoms compatible with polymyalgia rheumatica (PMR), and 23 (27.4%) had visual loss. Three (3.6%) patients had solid organ malignancies while two had hematologic malignancies (2.4%) before GCA diagnosis. TAB was obtained in 63 (75%) patients, in 47 of whom (74.6%) the pathological findings were consistent with GCA. A PET/CT scan has been performed before glucocorticoids (GCs) initiation in 43 (51.2%) patients and of these, 37 (86.0%) revealed uptake consistent with large vessel involvement. The median follow-up time of the 51 patients was 3.7 (IQR: 1.8–6.8) years. GCs were started promptly after the diagnosis. During the follow-up period, 28 (54.9%) patients experienced a relapse. Thirty-nine (78%) patients were under GC treatment, with a mean dosage of 4.8 ± 2.8 g/day at the final visit. At the final visit, 20.3% (17:84) had died whereas 9.8% (5:51) had permanent vision loss.

**Conclusion:**

Treatment of GCA is challenging. GCA causes serious morbidities and increased mortality. PET/CT is highly effective in detecting large vessel vasculitis in GCA and could perhaps replace TAB in the future.

## 1. Introduction

Giant cell arteritis is a large vessel vasculitis which may cause significant morbidity such as blindness and stroke [1]. The disease affects predominantly cranial arteries derived from the carotid arteries. The etiology of GCA is not clearly understood. T-cells, macrophages, and giant cells can be seen along with granulomatous lesions while histopathology disclosed intimal hyperplasia and destruction of elastic fibers in involved vessel wall [2].

GCA predominantly affects European populations especially those of Scandinavian origin (>20/100,000 people older than 50 years) [3,4] in comparison with the Southern European and Mediterranean races (Turkey and Tunisia: ~1.13–7/100,000 people older than 50 years) [5–8].

GCA cases usually present with cranial ischemic manifestations such as headache, visual symptoms, jaw claudication, and tense and swollen temporal arteries. Some may present with symptoms compatible with polymyalgia rheumatica (PMR) as well [9].

The diagnosis of the GCA should be based on typical clinical findings, elevated acute phase reactants, panarteritis in temporal artery biopsy (TAB), and large vessel involvement in imaging modalities [10,11]. Recently, PET-CT, a noninvasive technique, offers considerable benefit in the diagnosis of GCA with high sensitivity and specificity rates compared to other radiographical imaging modalities [12,13].

Glucocorticoids (GCs) are the mainstay in the management in GCA. Relapses occur frequently during the disease course [14]; therefore, the cumulative amount of the GCs is increased. Chronic use of GCs increases the risk of numerous comorbid conditions, including avascular necrosis, osteoporosis, fracture, infections, and cardiovascular disease. Large-vessel manifestations are well defined and include permanent vision loss, cranial arterial occlusions, stroke, aortitis, aortic dilatation/aneurysms or aortic dissection [15]. Although previous studies reported that mortality rates of GCA patients are similar to that found among healthy population [16,17], more recent studies disclosed an increased mortality especially in the first years of disease after the diagnosis [18,19]. Indeed, two different metaanalyses which underlined the mortality rates of GCA patients are similar to general population [20,21].

We evaluated presentation characteristics, diagnostic features, clinical outcomes, relapse frequency, and side effects of GC therapy among patients with GCA followed in a single tertiary institution.

## 2. Materials and methods

We identified 84 patients (41 M/43 F) who were diagnosed with GCA at the Cerrahpaşa Medical Faculty Rheumatology Department at İstanbul University-Cerrahpaşa, between April 1990 and December 2020. The diagnosis of GCA was made between 1990 and 2000 in 8 (9.5%), between 2000 and 2010 in 13 (15.5%), and between 2010 and 2020 in 63 (75%) patients. Seventy-five patients (89.3%) had fulfilled the inclusion criteria of 1990 American College of Rheumatology for GCA [10]. The remaining nine (11.7%) patients fulfilled two diagnostic criteria and additional active vasculitis features. Three of these nine had presented with acute vision loss in whom fundoscopic examination revealed arteritic ischemic optic neuropathy. The remaining eight patients had elevated acute phase reactants and signs of active large vessel vasculitis in the positron emission tomography-computed tomography (PET-CT) scan. PET-CT was started to be used routinely after 2010 in our institution. Clinical features and laboratory data as well as demographics, comorbid conditions (diabetes mellitus, hypertension), presentation symptoms, physical examination and histopathologic findings of the 84 patients were retrieved from digital medical records and hard-copy patient files. Of these 84 patients, 33 (39.3%) had a follow-up period of less than 12 months while the remaining 51 (60.7%) had been regularly followed more than a year at the outpatient clinic. Treatment modalities, relapses, and outcomes were analyzed in these 51 (24 M/27 F) patients.

Relapse was defined similar to what had been published previously [14], the patient status that required an increase in the drug doses or switching to a new therapy, and was classified in the following groups: a) a new or preexisting sign/symptom with simultaneous increase of either CRP or ESR was observed, b) a new or preexisting sign/symptom without any increase in acute phase reactants, and c) acute phase reactants were increased alone without any new signs/symptoms of GCA or another organic etiology. The biochemically proven relapse was defined by at least one of the following conditions [14]: CRP level > 5mg/L (normal: 0–5 mg/L) and/or ESR by the Westergren method >22 mm/h for men and >29 mm/h for women. Deaths were examined by Turkish Death Notification System^1^, a web-based software of Turkish Ministry of Health.

### 2.1. Statistical analysis

The normality of data was assessed using the Shapiro–Wilk test. Median values (25th percentile–75th percentile) were used for analysis of nonparametric data; Student’s t-test was performed for parametric data.

Chi-squared test was used for categorical variables. A p-value less than 0.05 was considered significant. The survival rate was analyzed using the Kaplan–Meier plot. Statistical analysis was performed with SPSS 20.0 software (IBM Corp., Armonk, NY, USA).

## 3. Results

A total of 84 patients (41 M/43 F) were included in the cohort. The mean age at diagnosis was 68.4 ± 7.9 years (range: 49–85). Clinical and laboratory manifestations at presentation are presented in Table 1.

### 3.1. Clinical characteristics at presentation and diagnostic modalities among 84 patients

**Figure 1:**
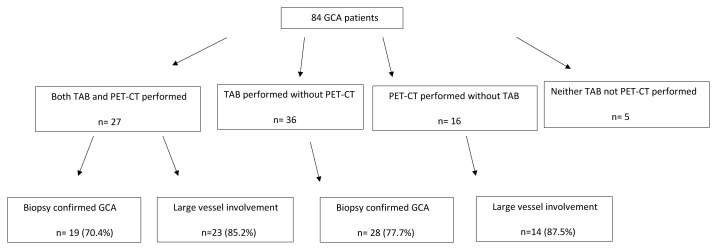
Diagnostic performances of the temporal artery biopsy and PET-CT in 84 GCA patients, TAB temporal artery biopsy, PET-CT positron emission tomography.

Sixty patients (71.4%) had presented with headache which was the leading initial complaint. Twenty-three (27.4%) had visual loss, 20 (23.8%) had jaw claudication, 19 (22.6%) had tenderness on the temporal area, and 22 (26.2%) had PMR symptoms. Twenty-five patients (29.8%) had fever, of whom 4 (16%) were diagnosed with fever of unknown origin at first. TAB was obtained in 63 (75%) patients, in 47 of whom (74.6%) the pathological findings were consistent with GCA. PET/CT scan was performed before GCs initiation in 43 (52.4%) patients and disclosed signs of diffuse large vessel vasculitis in 37 (86.0%) of these patients while aortic involvement was observed in 26 (60.5%) patients. Diagnostic performances of the TAB and PET-CT in the present study are depicted in Figure 1.

### 3.2. Comorbid conditions among 84 patients

Hypertension and diabetes mellitus were observed at the time of GCA diagnosis in 42 (50%) and 22 (26.2%) patients, respectively. Three (3.6%) patients had solid organ malignancies while two had hematologic malignancies (2.4%) before GCA diagnosis. Diagnosis of GCA and chronic myelomonocytic leukemia was concomitantly found in one patient, as we have previously reported [22].

### 3.3. Acute phase response, medical treatment and its complications among 51 patients

As depicted in Table 2, we evaluated treatment modalities and outcomes in 51 patients (24 M/27 F) who were regularly followed for at least 12 months. Median duration of follow-up of was 3.7 (IQR: 1.8–6.8) years. The median values of ESR and CRP at the diagnosis were 91.6 mm/h (IQR: 68.7–112.7) and 60 mg/L (25–111.5), respectively. These were found to be significantly lower by the time of final evaluation (ESR: 12.5 mm/h; IQR: 9–27.5 and CRP: 3 mg/L; IQR: 1.3–6.2) (for both ESR and CRP, p = 0.001).

GCs were initiated promptly following the diagnosis in all patients except one. Pulses of GC were preferred in 12 (23.5%) patients and oral GCs were used in the remaining 38 (76.5%) patients with a daily mean 45.8 ± 21.3 mg GC treatment. GCs were tapered to <5 mg/day in 28 (55.1%) patients within the first year, in 14 (28.5%) within the second year, and in 8 (16.3%) within the 5 years since the initial visit. After the initiation of GCs, the median time for tapering GC ≤5 mg/day was 8 months (IQR: 5–14.5 months). In 11 (22%) patients, GC therapy was terminated after a median duration of 23 months (IQR: 16.7–39 months) while 39 (78%) patients were under GC therapy with a mean duration of 4.8 ± 2.8 g/day at the final visit. Azathioprine (AZA) and methotrexate (MTX) were started in addition to GC while tapering GCs in 5 (10%) and 22 (43.1%) patients, respectively. Two patients (2:5) had to stop AZA because of gastrointestinal intolerance and pulmonary aspergillosis. The remaining 3 patients were still receiving AZA. MTX was discontinued in nine patients (6:22; 27.3%) due to the gastrointestinal intolerance (n = 2), myelosuppression (n = 2), pneumonitis (n = 1), genital herpes simplex infection (n = 1) and alopecia (n = 1). Moreover, in three patients (3:22) MTX was stopped after clinical and biochemical remission (Table 3). The remaining 12 patients were still receiving MTX.

A total of 9 patients were treated with tocilizumab (TCZ) after 2014. TCZ was given to eight GC-dependent patients after their first flare and to one patient who could not continue 1 mg/kg daily GCs because of the patient’s severe diabetic condition. Except one, clinical remission and laboratory improvement was observed in these patients during the TCZ therapy (Table 3). GCs were safely lowered during the TCZ treatment and four of these patients were able to stop using GCs at the final visit (Table 3). Seven of these nine patients were still receiving TCZ. One patient who had sustained remission with TCZ for 44 months was discontinued the therapy and relapse did not occurr after 1 year follow-up. The patient who had relapse with TCZ was switched to 100 mg daily anakinra, after 6 months during which he received TCZ, however, continued to have relapses. This patient responded to anakinra injections quickly and remained in remission for 4 years. Currently, he is asymptomatic and is receiving anakinra 100 mg every other day.

GC-associated osteoporosis, hypertension, and diabetes were observed in 11 (21.6%), 8 (15.7%) and 4 (%7.8) patients, respectively. Additionally, two patients (3.8%) had genital herpes, and two were admitted for pneumonia during the treatment course that turned into nocardiosis and aspergillosis. One patient had cutaneous herpes zoster during the TCZ treatment.

### 3.4. Flares and outcomes in 51 patients

Among 51 patients who were regularly followed, 15 (29.4%) had vision loss, 14 (27.4%) presented with PMR, and 25 (50.1%) of all 26 patients who were evaluated with PET-CT had large vessel vasculitis at initial visit. By the time of final evaluation, of the 15 patients with initial vision loss, five (35.7%) had complete remission, four (28.6%) had a partial remission, whereas five (35.7%) had permanent vision loss (one bilateral, four unilateral), and the remaining one patient was lost to follow-up due to complications following hip fracture and therefore could not be evaluated.

During a median follow-up of 3.7 years, a total of 28 patients (54.9%) relapsed. Twenty-five patients (49.0%) had one relapse, one had two relapses, and two had three relapses. Fourteen (50.0%) patients relapsed within the first year following the diagnosis, nine (32.1%) within the second and third years, and five (17.9%) patients within fourth and fifth years. Nineteen of the first relapses (76.0%) were attributed to only laboratory abnormalities, three (6.0%) were diagnosed with positive symptoms only, and six (12.0%) were due to the presence of abnormal laboratory studies in the setting of positive symptoms. Of these 28 patients who relapsed, four were not using GC while the remaining 24 were using GCs with a mean dose of 5.0 ± 3.2 mg/day.

### 3.5. Survival rate of the 84 patients

**Figure 2:**
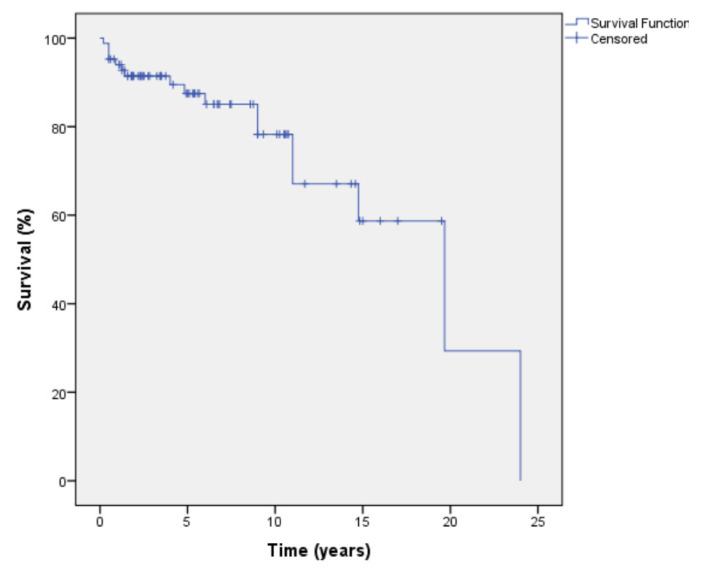
Kaplan–Meier survival plot of the 84 GCA patients*. *The exact dates of death were not determined in 7 out of 17 patients; therefore, these seven patients’ last visit dates were accepted as exact time of death.

By the end of the analysis, 17 (20.3%) of 84 patients had died. We could not determine the exact time of death in seven of these 17 patients. The remaining 10 patients had died after median 8.5 (IQR: 0.9–14.7) years of follow-up, while mortality causes were not determined. The survival rate of the 84 GCA patients is depicted in Figure 2.

## 4. Discussion

In this study, we evaluated the clinical manifestations, relapse rates, and outcomes of GCA in 84 patients (41 M/43 F) diagnosed and followed between 1990 and 2020. Our study suggests PET-CT is a valuable vascular imaging modality in GCA and highly effective (37:43, 86.0%) in detecting large vessel vasculitis.

It is well known that the most frequent clinical features of GCA are headache, ischemic manifestations, PMR, and temporal artery swelling [15]. While most of the clinical manifestations observed in the present study were similar with the reported series in the literature [16], some clinical features were relatively different. The frequency of vision loss in our study (27.4%) was in line with that reported previously (23%–28%) [23–25]. On the other hand, we observed a considerably lower frequency (26.2%) of PMR compared to other series (40%–73.7%) [8,9,26]. Deafness or stroke was not observed in any patients during the present study; in fact, these were well-defined intracranial manifestations of the disease [27]. Also, limb claudication was not observed as an initial presenting symptom which is also a rare but well-defined symptom [15]. These differences could be due to several reasons: a) a considerable portion of the patients was lost to follow-up in our series which may have impacted our results, b) the frequency and severity of GCA could be decreased in the Mediterranean region, c) those who presented with stroke, deafness, or other neurologic complaints had been taken care elsewhere and hence are not referred to rheumatology properly.

TAB used to be the gold standard in the diagnosis of GCA [28]; however, there are a number of problems with it, as previously reported: a) False-negative results with TAB are not rare [29,30], b) there are technical issues such as the length and localization [31–33], c) the timing of the biopsy after treatment initiation is also a controversial issue [34,35], and d) the necessity also is argued since it is an invasive procedure [36]. This situation emphasized the need of more accurate imaging modalities for the diagnosis. PET-CT was found to be more accurate for detecting large vessel involvement. Moreover, it could be able to differentiate malignancy in elderly patients who presented with constitutional symptoms. A metaanalysis disclosed that the sensitivity and specificity of PET-CT in GCA diagnosis was 90% and 98% respectively [37]. In our study, the sensitivity of the PET-CT was found as 86.0% (37:43) and that of TAB was calculated as 74.6% (47:63) which were in line with what had been reported previously.

The aortic aneurysm/dissection was the most fearful complication during the disease course. In a prospective study, of the 54 GCA patients who had biopsy-proven GCA, 22.2% had aortic dilatation/aneurysm during the follow-up [38]. Another retrospective study in 130 GCA patients revealed that those who had vascular FDG uptake at initial PET-CT were more prone to develop aortic complications during the follow-up [39]. These data indicate that screening GCA patients initially with PET-CT would be useful in order to estimate large vessel complications during the follow-up. In our current study, although our sample size was small and duration of follow-up was rather short, we did not observe any aortic aneurysm or dissection in those patients who had large involvement in their PET-CT scan.

Although GCs are the mainstay therapy for GCA, their starting dose, the rate of reduction, and total duration of therapy are not standardized. GC-related side effects are troublesome and occur frequently during the follow-up. In our study, osteoporosis was the most common GC-related side effect similar to previous reports [12,40].

There is an ongoing debate whether there is a link between GCA and malignancy. Ungprasert et al. disclosed a notably increased risk of malignancy among the GCA patients in a metaanalysis [41]. However, a recent Swedish population-based study showed that there was no increased risk of malignancy except leukemia [42]. Additionally, Mekinian et al. showed that systemic vasculitis was significantly more common than the other autoimmune or inflammatory disorders among the chronic myelomonocytic leukemia patients [43]. In the present study, we also observed considerable number of patients with concomitant solid organ or hematologic malignancies prior to GCA diagnosis.

The Giant Cell Arteritis Actemra (GiACTA) trial showed the efficacy of TCZ in patients with GCA [11]. In the current study, eight steroid-dependent patients were treated with TCZ. Eight of these patients received TCZ after their first flare. Additionally, one severe diabetic patient was treated with TCZ plus low dose GCs. Except one, all were successfully treated with TCZ. The one who relapsed under TCZ was switched to anakinra. Since then, the patient remained in sustained remission. Anakinra has limited data in the literature; it has been shown to provide remission in 3 patients who had been refractory to conventional treatment [44].

Published data on mortality among patients with GCA reveals slight increase of mortality rate in this patient population compared to normal population especially in the first years after the GCA diagnosis [18,19]. Ben Shabat et al. showed that the mortality was significantly increased over the first 2 years after the diagnosis particularly among those £70 years [ 18 ]. However, survival rate was not found to be impaired with longer follow-up of GCA [45]. An increased mortality rate was significantly associated with large vessel involvement [46]. In our study, we found that the mortality rate was 20.3% (17:84). Yet, we think the mortality rate we found should not be underestimated.

This study has several limitations. The number of patients who were lost to follow-up or those with a follow-up duration shorter than 12 months was large. We were unable to determine the exact time of death or the cause of death. Lack of standardization of diagnostic modalities was another limitation of the study. Moreover, since other related departments such as ophthalmology and neurology did not participate in the study, our study might not have represented the true number of causalities.

In conclusion, PET/CT is highly effective in detecting large vessel vasculitis in GCA and could perhaps replace TAB in the future. Side effects of GCs are frequent. Moreover, patients frequently had side effects during the MTX or AZA treatments. Biological treatment seems to be beneficial, and its widespread use should be encouraged. Finally, GCA is a serious disease with significant morbidity and mortality.

## Figures and Tables

**Table 1:** Demographic and clinical characteristics at presentation and laboratory manifestations among 84 patients.

Age, mean **±** SD, years	68.4 ± 7.9
Male, n (%)	41 (48.9)
Fulfilled ^3^3 GCA ACR criteria, n (%)	75 (89.3)
Temporal artery biopsy, n (%)	63 (75)
Biopsy–proven GCA, n (%)	47 (74.6)
PET-CT scan, n (%)	44 (52.6)
PET-CT–proven large vessel vasculitis, n(%)	38 (86.4)
*Comorbidities, n (%)*	
Diabetes	22 (26.2)
Hypertension	42 (50)
*Clinical symptoms, n (%)*	
Headache	60 (71.4)
Jaw claudication	20 (23.8)
PMR	22 (26.2)
Vision loss	23 (27.4)
Fever	25 (29.8)
*Laboratory*	
ESR, median (IQR), mm/h	91.6 (68.7–112.7)
CRP, median (IQR), mg/L	60 (25–111.5)

**Table 2:** Treatment modalities and outcomes of the 51 patients.

Characteristic	Value
*Follow-up duration, median (IQR), years*	3.7 (1.8–6.8)
*Initial treatment*	
Pulse dose steroid, n (%)	12 (23.3)
Initial prednisone dose, mean (S.D), mg/day	45.8 ± 21.3
*GCs tapered to <5 mg/day, n (%)*	
within 1st year	28 (55.1)
within 2nd year	14 (28.5)
within until 5 years	8 (16.3)
*Relapse, n (%)*	
no relapse	23 (45.1)
one relapse	25 (49.0)
two relapses	1 (2.0)
three relapses	2 (3.9)
Vision loss, n (%)*	15 (29.4)
Outcome of the vision loss after the treatment, n (%)	
complete remission	5 (35.7)
partial remission	4 (28.6)
permanent vision loss	5 (35.7)

* One patient was lost to follow-up due to complications following hip fracture and therefore could not be evaluated.

**Table 3:** Clinical and laboratory characteristics of the GCA patients prior and post TCZ therapy.

Characteristic	Pre-TCZ (n = 9)	Post-TCZ (n = 9)	p-value
Age at diagnosis, median (IQR), years	67 (65–69.5)		N/A
Disease duration prior TCZ, median (IQR), months	16 (4.5–18)		N/A
Symptoms, n (%)			
Constitutional symptoms	7 (77.8)	0	N/A
Headache	1 (11.1)	0	N/A
Any visual disturbance	1 (11.1)	1 (11.1)	0.99
Comorbidities, n (%)			
Hypertension	5 (55.6)		N/A
Diabetes	6 (66.7)		N/A
Laboratory			
CRP, median (IQR), mg/L	10 (4.4–22)	2.9 (0.3–2.1)	0.02
ESR, median (IQR), mm/h	46 (21–59)	9 (4–14)	0.001
Prednisolone use, n (%)	9 (100)	5 (55.5)	N/A
Prednisolone dose, median (IQR), mg/day	15 (8.7–17.5)	4 (2.5–12.5)	0.001
Methotrexate use, n (%)	4 (44.4)	1 (11.1)	0.11

*GCA* giant cell arteritis, *TCZ* tociluzumab, *ESR* erythrocyte sedimentation rate, CRP c-reactive protein

## Footnotes

1 T.C Sağlık Bakanlığı Ölüm Bildirim Sistemi [online] https://obs.saglik.gov.tr/ [accessed 1 September 2021]
